# Adaptive Algorithms as Control Strategies of Smart Upper Limb Orthosis: A Protocol for a Systematic Scoping Review

**DOI:** 10.3389/fnins.2021.660141

**Published:** 2021-05-07

**Authors:** Ledycnarf J. Holanda, Ana Paula M. Fernandes, Júlia A. de Amorim, Aryel M. Matias, Severino P. Nunes Netto, Danilo A. P. Nagem, Ricardo A. de M. Valentim, Edgard Morya, Ana Raquel Lindquist

**Affiliations:** ^1^Laboratory of Intervention and Analysis of Movement, Department of Physical Therapy, Federal University of Rio Grande do Norte, Natal, Brazil; ^2^Laboratory of Technological Innovation in Health, Federal University of Rio Grande do Norte, Natal, Brazil; ^3^Department of Biomedical Engineering, Federal University of Rio Grande do Norte, Natal, Brazil; ^4^Edmond and Lily Safra International Institute of Neurosciences, Santos Dumont Institute, Macaiba, Brazil

**Keywords:** adaptive algorithms, upper limb orthosis, upper limb rehabilitation, functionality, movement

## Abstract

**Introduction:** Adaptive algorithms for controlling orthosis emerged to overcome significant problems with automatic biosignal classification and personalized rehabilitation. Smart orthoses are evolving fast and need a better human-machine interaction performance since biosignals, feedback, and motor control dynamically change and must be adaptive. This manuscript outlines a scoping review protocol to systematically review the smart upper limb (UL) orthoses based on adaptive algorithms and feasibility tests.

**Materials and Methods:** This protocol was developed based on the York framework. A field-specific structure was defined to achieve each phase. Eleven scientific databases (PubMed, Web of Science, SciELO, Koreamed, Jstage, AMED, CENTRAL, PEDro, IEEE, Scopus, and Arxiv) and five patent databases (Patentscope, Patentlens, Google Patents, Kripis, J-platpat) were searched. The developed framework will extract data (i.e., orthosis description, adaptive algorithms, tools used in the usability test, and benefits to the general population) from the selected studies using a rigorous approach. Data will be described quantitatively using frequency and trend analysis methods. Heterogeneity between the included studies will be assessed using the Chi-test and I-statistic. The risk of bias will be summarized using the latest Prediction Model Study Risk of Bias Assessment Tool.

**Discussion:** This review will identify, map, and synthesize the advances about the description of adaptive algorithms for control strategies of smart UL orthosis using data extracted from patents and articles.

## 1. Introduction

Accurate motor control is critical for functioning orthosis in daily living (Sengur et al., 2017; Trigili et al., 2019; Arteaga et al., 2020). There are several orthosis controllers, but extracting data to improve its performance is still a computational limitation (Chemuturi et al., 2013). Algorithms that enable patient-orthosis interface to daily use will improve the control of voluntary movements and functional rehabilitation.

### 1.1. Adaptive Algorithms

The adaptive control consists of a system that could adapt and predict performance in real-time during movements with high accuracy (Hasan and Dhingra, 2021). This type of system can regulate the operation of the interface, from the operating parameters extracted in real-time to reach the best mode of performance (Ljung et al., 2012; Hasan and Dhingra, 2021). Adaptive algorithms can automatically adjust the parameters to categorize individual movement patterns with fewer data and hardware requirements (Chemuturi et al., 2013; Allard et al., 2016).

The user-centered design is one of the requirements of hardware and new technologies development (Manna and Bhaumik, 2013; Gupta et al., 2020). As the current devices are not sufficiently safe to operate physically with people with movement disorders (Chandrasiri et al., 2019), it is required that the system is adaptable to different individuals (Cheung et al., 2017) in order to avoid uncomfortable or unnatural posture, and has multiple degrees of freedom to provide better rehabilitation results (Gupta et al., 2020).

Adaptive algorithms and robot-aided upper limb (UL) rehabilitation must guarantee active training management, patient assistance if necessary (Gupta et al., 2020), and quantify residual muscle strength levels, spasticity, fatigue (Yousif et al., 2019; Bashford et al., 2020), or brain activity (Delijorge et al., 2020). Current studies have shown that exoskeletons can assist in highly repetitive UL task-oriented movement training, and improve daily living activities (Mao and Agrawal, 2011; Sengur et al., 2017; Trigili et al., 2019). Thus, robust methods to automatically classify and identify the UL movement patterns can improve exoskeleton control (Arteaga et al., 2020).

### 1.2. Smart Orthoses

Chu and Patterson (2018) observed that both the design and feedback of many devices need to be improved to maximize patient safety and rehabilitation outcomes. Previous researches (Proietti et al., 2016) have focused on increment this type of device with adaptive algorithms. Moreover, its impact on motor performance needs to be assessed to ensure its usability in individuals with different disorders. Physical (e.g., appearance, weight, and size) and ergonomic features must be considered because they interfere with functionality and affect performance (Merchant et al., 2018).

Successful rehabilitation relies on patient's ability to participate in therapeutic activities and can be influenced by individualized task prescription, considering frequency, duration, and therapy costs. However, passive training is not adequate to recover motor functions. Patient's active training is related to neuroplasticity function (Perez-Ibarra et al., 2018). When devices are properly applied, they can provide better results than conventional approaches, including standardized training, adaptation to loads, and prevention of muscle atrophy (Wu and Chen, 2020).

Smart orthoses based on adaptive algorithms may replicate exercise, and support tools to improve rehabilitation, compliance, and outcomes. From this perspective, we aim to perform the first review related to smart orthoses based on adaptive algorithms and compare usability, algorithms, designs, and benefits between different tools to gather evidence to develop these devices. Therefore, a detailed systematic scoping review will support developers and rehabilitation professionals, through providing answers to the following questions: What mechatronic architectures and characteristics of the systems were used to develop UL orthoses? What adaptive algorithms were used in the orthoses? What tools were used to test orthosis usability? What benefits were found in individuals who have used orthosis?

This paper outlines a scoping review protocol to systematically review the smart UL orthosis based on adaptive algorithms. We will examine the design description, operation, usability test, and registered patent. Eligibility criteria were also defined to include different studies and investigate how UL orthoses are being currently developed. Systematic scoping reviews offer feasible methods for collecting and synthesizing a wide range of evidence (Peters et al., 2015b) and are particularly useful for bringing together evidence from different sources. Information gathered will allow us to correlate published studies with registered patents and propose recommendations to guide new orthoses based on adaptive algorithms.

## 2. Materials and Methods

### 2.1. Protocol Design

A scoping review will be designed to identify, analyze gaps, and obtain an overview of the emerging evidence to make clearer what can be investigated in a more appropriate way by a systematic review (Munn et al., 2018). This protocol is based on the York framework proposed by Arksey and O'Malley (2005) that includes five phases: identifying the research question (1), identifying relevant studies (2), study selection (3), charting the data (4), and collating, summarizing and reporting the results (5).

In addition, the Joanna Briggs Institute (JBI) (Peters et al., 2015a) framework will be used to clarify and provide a greater relationship between the title, review objective(s), question(s), and inclusion criteria. Thus, a field-specific structure based on an initial exploration of studies and aspects related to orthosis description, device operation, control system characteristics, acquisition algorithm and its processing techniques, adaptive algorithm parameters, characterization of the participants, intervention description, and results of each study, will guide each phase of the review.

The present protocol is reported according to the Preferred Reporting Items for Systematic reviews and Meta-Analyses (PRISMA-P) protocols (Moher et al., 2015) as suggested in the PRISMA Extension for Scoping Review (PRISMA-ScR) guidelines (Tricco et al., 2018) (see checklist in Supplementary File 1), and its updated checklist (Rethlefsen et al., 2021) (see updated checklist in Supplementary File 2).

### 2.2. Stage 1: Identifying the Research Question

This review will explore specific questions such as feasibility, orthosis effectiveness, and orthosis characteristics and concepts (Munn et al., 2018). Therefore, the review questions were previously defined to provide the roadmap for subsequent stages.

During the consultation with our systematic scoping review experts (EM, ARRL, RAMV, and DAPN), a set of questions were elaborated for each of the following aspects (Table 1): (1) description of both the orthosis (2) and the adaptive algorithms used, (3) tools used to test usability, and (4) benefits for the general population.

**Table 1:** Research questions.

**Aspects**	**List of questions**
Description of orthoses	•What mechatronic architecture was used to develop orthosis for UL standard rehabilitation? •What are the characteristics of the systems used to control the orthosis?
Description of the adaptive algorithms used	•What adaptive algorithms were used in the orthosis for UL rehabilitation?
Tools used for the usability test	•What tools were used to test orthosis usability?
Benefits when using orthosis	•What benefits were found in individuals who have used orthosis?
Correlation between patents and articles	•How many included patents were published in scientific journals?

It is worth mentioning that the research questions of this study will not be limited to those presented in Table 1. Further questions can be discussed based on data analyses at the systematic scoping review elaboration.

The JBI suggests using the Population, Concept, and Context (PCC) to construct scope review questions since it is a less restrictive alternative to the PICO (Peters et al., 2015a).

The target “population” will include people aged ≥18 years, regardless of gender, and health status (i.e., healthy or unhealthy with acute or chronic diseases). The “concept” will cover all studies that developed smart orthoses based on adaptive algorithms. The “context” will cover comparisons among mechatronic architecture, algorithms, and types of human-machine interface controller. We will also compare the impact of UL orthosis controlled by adaptive algorithms on motor function and physical therapy treatment.

### 2.3. Stage 2: Identifying Relevant Studies

Systematic scoping reviews provide a large area of scientific evidence on a particular topic. The search strategy elaboration using keywords and synonyms based on the Medical Subject Headings (MeSH) must also be broad and will consider four significant areas of the theme: body segment, orthosis, physical rehabilitation, and adaptive algorithms. Two sections will be created to describe the steps for selecting the relevant studies: search resources and search strings.

#### 2.3.1. Search Resources

To conduct a comprehensive search, the York framework recommends searching several literature sources, including electronic databases, reference lists of relevant literature, a manual search of key journals, conference proceedings presenting relevant publications regarding the review topic, and patent websites. Numerous keywords were combined to formulate the search strings (Table 2).

**Table 2:** Main concepts and related keywords.

**Concept**	**Matching keywords**
Body segment	•Hand, Elbow, Wrist, Shoulder, Forearm, Arm, Fingers, Upper Extremity, Upper Limb.
Orthosis	•Orthosis, Orthoses, Active Orthosis, Active Orthoses, Exoskeleton, Orthotic Device, Orthosis Device, Orthoses Device, Robotic Device, Robotic, Wearable Robot, Exosuit, Wearable Orthoses, Wearable Orthosis, Wearable Assistive Robots, Wearable Exosuit.
Physical rehabilitation	•Physical Rehabilitation, Motor Rehabilitation, Physical Medicine, Telerehabilitation, Physical Therapy, Functional Orthoses, Functional Outcome.
Adaptive algorithms	•Machine Learning, Supervised Machine Learning, Unsupervised Machine Learning, Semi-Supervised Machine Learning, Reinforcement Learning, Artificial Intelligence, Adaptive Algorithms, Neural Network, Artificial Neural Network, K-Nearest Neighbors, Linear Regression, Logistic Regression, Support Vector Machines, Decision Trees, Random Forests, Extreme Gradient Boosting, K-Means, Hierarchical Cluster Analysis, Expectation Maximization, Principal Component Analysis, Kernel Principal Component Analysis, Locally-Linear Embedding, T-Distributed Stochastic Neighbor Embedding, Q-Learning, State-Action-Reward-State-Action, Deep Q Network, Deep Deterministic Policy Gradient, Computational Intelligence, Machine Intelligence, Computer Reasoning, Computer Vision Systems, Knowledge Acquisition, Knowledge Representation, Logic Fuzzy, Fuzzy Control, Deep Learning.

From this perspective, the search in databases and patent websites will be divided into several steps:

1. A comprehensive search will be conducted in the PubMed, Web of Science, SciELO, Koreamed, Jstage, AMED, CENTRAL, PEDro, IEEE, Scopus, and Arxiv databases; as well as the Patentscope, Patentlens, Google Patents Kripis, and J-platpat websites. These sources include highly important journals related to the areas of this scoping review (health and medicine, information science and technology, engineering, and computer science).
2. The reference list of all included studies and patents will be manually checked to search for additional relevant studies.

#### 2.3.2. Search Strings

Our multidisciplinary team planned a list of pertinent terms for both the databases and patents (Table 2). We plan to conduct a sensitive rather than specific search of the literature. Thus, the search terms will be kept broad, and irrelevant studies will be eliminated in the study selection phase.

All articles searches will be performed without language restriction or the publication year (see all search strategies for all database in Supplementary File 3).

Regarding the patent, searches will be conducted in the English, Spanish, French, Japanese and Korean and German languages, and those patents with <10 years will be retained (see all search strategies for all patents websites in Supplementary File 4).

### 2.4. Stage 3: Study Selection

The inclusion criteria for selecting the articles will be:

1. Types of studies: We will include full-text studies and experimental study designs, including randomized controlled trials (RCTs). In the absence of RCTs, non-randomized controlled trials, quasi-experimental, before and after studies, prospective and retrospective cohort studies, case-control studies, and analytical cross-sectional studies will be included. This review will also consider descriptive epidemiological study designs, including case series, individual case reports, and descriptive cross-sectional studies for inclusion. We will exclude review studies, book chapters, and duplicate articles.
2. Types of participants: This review will consider studies that performed the usability test in human adults aged 18 years or over, either healthy or who have a clinical diagnosis of either any acute or chronic UL movement disorder with any degree of severity.
3. Types of interventions: This review will include all studies that developed smart UL orthosis based on adaptive algorithms and that did or did not perform the usability test. The usability test description should contain instrumented (optoelectronic systems, electrogoniometer, electromyography and/or inertial measurement unit sensors) and non-instrumented measurements (standard clinical assessments, questionnaires, and verbal reports) before and during device use.
4. Types of orthosis: This review will include studies that described the following items of the orthosis: materials, actuators, sensors, methods and sensor and actuator placements; device operation (microcontroller) and adaptive algorithms. Studies in which the device was intended for use as prostheses or passive orthosis will be excluded.

On the other hand, the criteria for selecting the patents are only about the types of orthoses similar to the one described above, and the following:

1. Year of publication: Patents registered in the last 10 years.
2. Language: Patents published in the English, Spanish, French, German, Japanese, and Korean.

We chose articles and patents published in several languages in the last 10 years to obtain an overview of the evidence about the adaptive algorithms used as a control strategy for UL orthoses. In this way, we will be able to guide developers of this type of orthosis and professionals who use it in rehabilitation.

#### 2.4.1. Screening Process

Before the screening process, two research team members (LJH, APMF) will conduct the inclusion and exclusion criteria within a random sample of 10% of the retrieved cases. Once the final set of criteria will be performed, the titles and abstracts, and/or previews will be simultaneously examined to search the relevant studies and patents. For this, four authors (LJH, APMF, JAA, SPNN) will independently apply the inclusion criteria on all retrieved citations, and the full-text of the included articles and patents will be analyzed in the second phase for final inclusion decision. Disagreements will be resolved by consensus with the fourth reviewer (SPNN).

According to the JBI guideline (Peters et al., 2015a), the screening process will be reported in a graphical diagram similar to the Preferred Reporting Items for Systematic Reviews and Meta-Analyses (PRISMA) chart (adapted for scoping reviews). The flowchart will detail the review decision processes, including duplicate removal, the number of excluded articles, and the reason for exclusion.

### 2.5. Stage 4: Charting the Data

Five authors will construct a descriptive summary of the results: LJH and APMF will insert the article data, while APMF, JAA, and SPNN will insert the patent data. For this stage, a data charting form will be developed with variables corresponding to the research questions. The components of the identified frameworks will be used to determine an initial set of variables updated as “articles being reviewed.” A description of frameworks and their components is provided below and summarized in Table 3.

**Table 3:** List of variables to be studied for each aspect.

**Aspects**	**Variables**
Description of the orthosis	•What mechatronic architecture was used to develop the orthosis for UL rehabilitation in the general population? To answer this question, it might be considered whether the orthosis presents the following features: type of assisted motion, assisted body segment, the total degree of freedom, portability, safety, user intent modality, the number and types of actuators, weight, input force, external torque/grip force, power transmission, and device operation. •What were the system characteristics used to control the orthosis? The human-machine interface, the number of electrodes used, control strategy, cue type or go signal, supporting feedback, and both control type and action will be considered to answer this question.
Description of the adaptive algorithms used	•What types of adaptive algorithms were used in the orthosis for UL rehabilitation in the general population? To answer this question, the developed algorithm might contain the following information: metrics used for evaluating algorithms, types of adaptive algorithms, and input and output data.
Tools used to the orthosis usability test	•What tools were used to test its usability? To answer this question, it might be considered whether any instrumented or non-instrumented measurements to investigate motor performance, quality of life and user satisfaction were used. To make these benefits clearer, it is necessary to describe how the assessment was performed. If an intervention was performed, what were the frequency, duration, and description?
Benefits when using orthosis	•What benefits were found in individuals who have used orthosis? The sample size, diagnosis, diagnosis time, age, gender must be described to clarify these in each type of disease, such as orthopedic, rheumatological or neurological diagnosed in the people tested so that the benefits that the orthoses are capable of are clearer provide in each disease, as previously mentioned.
Correlation between patents and articles	•How many patents included in the review were published in scientific journals? Moreover, how many of these performed usability tests?

### 2.6. Stage 5: Collating, Summarizing, and Reporting the Results

The extracted data will be inserted in a table in which rows will represent the included articles and patents, columns will represent the variables, and cells will contain the strategies used for the relevant variables. To analyze this data set, frequency and trend analyses will be used. The risk of bias assessment and confidence in cumulative evidence will also be performed.

#### 2.6.1. Risk of Bias Assessment

Two authors (LJH and APMF) will perform the risk of bias assessment using qualitative analyzes. Disagreements will be resolved by consensus with the third reviewer (JAA). The Prediction model study Risk of Bias Assessment Tool (PROBAST), which includes 20 questions divided into four domains (participants, predictors, outcome, and analysis), will be used, and the risk of bias for each domain will be classified as low risk, high risk, or too unclear for judgment (Wolff et al., 2019).

#### 2.6.2. Confidence in Cumulative Evidence

Confidence in cumulative evidence will be performed in accordance with the Grading of Recommendations, Assessment, Development and Evaluations (GRADE) guideline (Schünemann et al., 2013). This system grades it at four levels: high, moderate, low, and very low.

## 3. Statistical Analysis and Data Synthesis

A meta-analysis will be carried out for the outcome measures when possible. We will search for study heterogeneity using the I^2^ statistic (Higgins and Thompson, 2002; Higgins et al., 2003), and Chi-squared test provided by the Cochrane statistical software Review Manager (RevMan, 2014). We will enter with studies sufficiently similar in clinical and methodological terms. Considering that adaptive algorithms are new in orthosis interface, we expect few studies. It means that the estimates of between-study variation (tau-squared) will be less robust, and in this case a random-effects model can be used to obtain pooled estimates, if the Chi-squared test is statistically significant with a *P* < 0.10, or if the I^2^ statistic is greater than 50%. We will guide our interpretation of I^2^ statistic suggested by RevMan (2014) and Deeks et al. (2019).

Besides, the reasons for heterogeneity (e.g., participants, interventions, orthosis types, algorithm types, and risk of bias) will also be explored. In this case, a meta-synthesis or narrative synthesis using the frequency analysis will be performed.

### 3.1. Trend Analysis

Trend analysis will be used to present the research evolution based on several variables and associations. The organization by clusters will combine the studies for data exploration (e.g., explore the performance of a specific machine learning technique and obtain specific findings), resulting in a map of studies represented as a bubble plot, graph, or table.

In this stage, two main aspects will be considered: (1) the demographic geographic characteristics in which the clustering will be used to define the patterns of the state of the art development and (2) correlations between the different centers in the world. Concomitantly, a regression analysis will be carried out to explore patterns concerning the development of the topic over time.

## 4. Discussion

To our knowledge, this will be the first review describing adaptive algorithms for control strategies of smart UL orthosis using data extracted from patents and articles to identify, map, and synthesize advances in this field. In this study, a detailed systematic scoping review protocol has been developed to conduct a comprehensive review within the field of orthoses and adaptive algorithms using the framework proposed by Arksey and O'Malley. Several enhancements were applied to the adopted framework to be used in orthosis based on adaptive algorithms. Future systematic scoping reviews will use our protocol as an enhanced version of previous frameworks with more details regarding extraction, categorization, and technique analyses.

These information will be important to guide multidisciplinary teams involved in different aspects, such as professionals with engineering expertise, who will develop the hardware and software components of the orthosis, and rehabilitation professionals, who can use this type of device as a therapeutic resource. Specifications about adaptive algorithms will be important to offer performance metrics for developed methods to offer an individualized and precise adjustment during the execution of the movement in routine activities and in clinical environments. Health professionals will also improve knowledge regarding UL orthosis to apply in clinical practice and home settings. This review will direct knowledge on how to improve the patient-orthosis interaction through hybrid systems capable of offering individualized and precise assistance for the needs of each individual, considering the level of functional capacity for the execution of each task, supporting the rehabilitation, functionality and quality of life.

## Author Contributions

LJH has conceptualized the review approach, has developed eligibility criteria and data extraction framework, which was then further developed by input from team members, and has elaborated the protocol framework. AMF has elaborated on the strategy search and has written this protocol. SPNN, AMM, and JAA have written this protocol. ARL, DAPN, RAMV, and EM were our experts on the subject of this review. All authors have made substantial intellectual contributions to the development of this protocol, and have read and approved the final protocol.

## Conflict of Interest

The authors declare that the research was conducted in the absence of any commercial or financial relationships that could be construed as a potential conflict of interest.
