# Supplementary material for: Adaptive Algorithms as Control Strategies of Smart Upper Limb Orthosis: A Protocol for a Systematic Scoping Review
Source: Front Neurosci. 2021 May 7;15:660141. doi: 10.3389/fnins.2021.660141 (PMC8138030; doi:10.3389/fnins.2021.660141)
Supplement: Supplementary file 2 [file Data_Sheet_2.PDF]

ADDITIONAL FILE 2 Updated PRISMA-ScR checklist

Section/topic	#	Checklist item	Location(s) Reported
**INFORMATION SOURCES AND METHODS**			
Database name	1	Name each individual database searched, stating the platform for each.	4
Multi-database searching	2	If databases were searched simultaneously on a single platform, state the name of the platform, listing all of the databases searched.	-
Study registries	3	List any study registries searched.	-
Online resources and browsing	4	Describe any online or print source purposefully searched or browsed (e.g., tables of contents, print conference proceedings, web sites), and how this was done.	4-5, Table 2
Citation searching	5	Indicate whether cited references or citing references were examined, and describe any methods used for locating cited/citing references (e.g., browsing reference lists, using a citation index, setting up email alerts for references citing included studies).	It will be done, as mentioned on Page 4
Contacts	6	Indicate whether additional studies or data were sought by contacting authors, experts, manufacturers, or others.	-
Other methods	7	Describe any additional information sources or search methods used.	-
**SEARCH STRATEGIES**			
Full search	8	Include the search strategies for each database and information source, copied and	Additional files

strategies		pasted exactly as run.	3 and 4
Limits and restrictions	9	Specify that no limits were used, or describe any limits or restrictions applied to a search (e.g., date or time period, language, study design) and provide justification for their use.	7
Search filters	10	Indicate whether published search filters were used (as originally designed or modified), and if so, cite the filter(s) used.	Only patents will be restricted in terms of year and language
Prior work	11	Indicate when search strategies from other literature reviews were adapted or reused for a substantive part or all of the search, citing the previous review(s).	5
Updates	12	Report the methods used to update the search(es) (e.g., rerunning searches, email alerts).	-
Dates of searches	13	For each search strategy, provide the date when the last search occurred.	It will be inserted in the final version of the systematic scoping review
**PEER REVIEW**			
Peer review	14	Describe any search peer review process.	Additional files 3 and 4
**MANAGING RECORDS**			

Total Records	15	Document the total number of records identified from each database and other information sources.	It will be inserted in PRISMA flowchart, as cited on P. 5
Deduplication	16	Describe the processes and any software used to deduplicate records from multiple database searches and other information sources.	Not applied

PRISMA-S: An Extension to the PRISMA Statement for Reporting Literature Searches in Systematic Reviews
Rethlefsen ML, Kirtley S, Waffenschmidt S, Ayala AP, Moher D, Page MJ, Koffel JB, PRISMA-S Group.
Last updated February 27, 2020.
