# Supplementary material for: Adaptive Algorithms as Control Strategies of Smart Upper Limb Orthosis: A Protocol for a Systematic Scoping Review
Source: Front Neurosci. 2021 May 7;15:660141. doi: 10.3389/fnins.2021.660141 (PMC8138030; doi:10.3389/fnins.2021.660141)
Supplement: Supplementary file 7 [file Table_3.DOCX]

**Appendix 2. Patents Search Scripts in Patent Scope.**

**Filters: Only patents registered in the last 10 years will be inserted.**

| Search Scripts |
| --- |
| (Hand OR Elbow OR Wrist OR Shoulder OR Arm OR Fingers OR Upper Extremity OR Upper Limb) AND (Orthosis OR Orthoses OR Exoskeleton OR Orthotic Device OR Orthosis Device OR Orthoses Device OR Robotic Device OR Robotic OR Wearable Robot OR Exosuit OR Wearable Orthoses OR Wearable Orthosis OR Wearable Assistive Robots OR Wearable Exosuit) AND (Physical Rehabilitation OR Motor Rehabilitation OR Physical Medicine OR Telerehabilitation) AND (Machine Learning OR Supervised Machine Learning OR Unsupervised Machine Learning OR Semi-Supervised Machine Learning OR Reinforcement Learning OR Artificial Intelligence OR Adaptive Algorithms OR Neural Network OR Artificial Neural Network OR K-Nearest Neighbors OR Linear Regression OR Logistic Regression OR Support Vector Machines OR Decision Trees OR Random Forests OR Extreme Gradient Boosting OR K-Means OR Hierarchical Cluster Analysis OR Expectation Maximization OR Principal Component Analysis OR Kernel Principal Component Analysis OR Locally-Linear Embedding OR T-Distributed Stochastic Neighbor Embedding OR Q-Learning OR State-Action-Reward-State-Action OR Deep Q Network OR Deep Deterministic Policy Gradient OR Computacional Intelligence OR Machine Intelligence OR Computer Reasoning OR Computer Vision Systems OR Knowledge Acquisition OR Knowledge Representation OR Logic Fuzzy OR Fuzzy Control OR Deep Learning) |
